# *Not a magic pill:* a qualitative exploration of provider perspectives on antibiotic prescribing in the outpatient setting

**DOI:** 10.1186/s12875-018-0788-4

**Published:** 2018-06-23

**Authors:** Traci D. Yates, Marion E. Davis, Yhenneko J. Taylor, Lisa Davidson, Crystal D. Connor, Katherine Buehler, Melanie D. Spencer

**Affiliations:** 1 Center for Outcomes Research and Evaluation, Atrium Health, Charlotte, NC USA; 2 Division of Infectious Disease, Atrium Health, Charlotte, NC USA

**Keywords:** Qualitative research, Antibiotic prescribing, Patient-provider relationships, Outpatient setting, Primary care, Antibiotic prescribing decisions, Patient antibiotic education

## Abstract

**Background:**

Inappropriate prescribing of antibiotics poses an urgent public health threat. Limited research has examined factors associated with antibiotic prescribing practices in outpatient settings. The goals of this study were to explore elements influencing provider decisions to prescribe antibiotics, identify provider recommendations for interventions to reduce inappropriate antibiotic use, and inform the clinical management of patients in the outpatient environment for infections that do not require antibiotics.

**Methods:**

This was a qualitative study using semi-structured interviews with key informants. Seventeen outpatient providers (10 medical doctors and 7 advanced care practitioners) within a large healthcare system in Charlotte, North Carolina, participated. Interviews were audio recorded, transcribed, and analyzed for themes.

**Results:**

Primary barriers to reducing inappropriate antibiotic prescribing included patient education and expectations, system-level factors, and time constraints. Providers indicated they would be interested in having system-wide, evidence-based guidelines to inform their prescribing decisions and that they would also be receptive to efforts to improve their awareness of their own prescribing practices. Results further suggested that providers experience a high demand for antibiotic prescriptions; consequently, patient education around appropriate use would be beneficial.

**Conclusions:**

Findings suggest that antibiotic prescribing in the outpatient setting is influenced by many pressures, including patient demand and patient satisfaction. Training on appropriate antibiotic prescribing, guideline-based decision support, feedback on prescribing practices, and patient education are recommended interventions to improve levels of appropriate prescribing.

## Background

Antibiotic resistant organisms and the infections they cause are an urgent public health threat worldwide [1]. Inappropriate antibiotic prescribing is the most significant factor behind increasing resistance that causes more than two million illnesses annually in the United States [1, 2]. Outpatient settings account for more than 154 million antibiotic prescriptions each year [3]. As much as 50% of antibiotics prescribed for acute respiratory infections and 30% of oral antibiotics prescribed across all conditions in the outpatient setting may be unnecessary or inappropriate [4]. This misuse of antibiotics is a major contributor to antibiotic resistant infections that cause over 23,000 deaths annually [5]. While several strategies to improve outpatient antibiotic prescribing have been proposed, challenges to the success of these interventions include variations across settings and geography, levels of clinician acceptance, and issues of sustainability [6–13].

Research on the specific factors that impact antibiotic prescribing practices in outpatient settings [4] and how patient-provider dynamics play a role in influencing care decisions made in those environments is limited. Given that most antibiotic prescriptions originate in the outpatient setting [14], these interactions may be crucial to clinical outcomes. Indeed, previous research has shown that physicians are more likely to prescribe medications inappropriately (e.g., opiates and antipsychotics) in the face of real or perceived expectations from patients [15–18]. If the same pattern is true for antibiotics in the outpatient setting, addressing these dynamics has the potential to improve practice and reduce inappropriate prescribing.

This study was part of a larger project focused on reducing inappropriate antibiotic prescribing in the outpatient setting. The goals of this study were to explore factors influencing provider decisions to prescribe antibiotics, identify provider recommendations for interventions to reduce inappropriate antibiotic use, and inform the clinical management of patients in the outpatient environment for infections that do not require antibiotics. This study adds to available evidence regarding how and why providers decide to prescribe antibiotics by incorporating the perspectives not only of physicians, but also of advanced care practitioners [19–21]. Moreover, it responds to a previous call for updated studies of antibiotic utilization among primary care providers within the United States [21].

## Methods

### Setting

This study was conducted at Atrium Health, a large, integrated healthcare network in the Southeastern United States. Atrium Health is a comprehensive, not-for-profit, healthcare organization comprised of over 900 care locations including hospitals, doctors’ offices, urgent care clinics, emergency departments, and long-term care facilities, with over twelve million patient encounters annually. This study focused on providers in outpatient primary care settings (i.e., doctors’ offices, urgent care clinics).

### Ethical considerations

The Atrium Health Institutional Review Board reviewed this study and classified it as quality improvement, therefore determining that project activities were not required to act in accordance with research policies and regulations. Still, an information sheet detailing the purpose of the study, how the study would work, and participants’ role in the research, including risks, benefits, reimbursement for time, costs, and confidentiality was reviewed at the beginning of each interview. Verbal consent to participate in the study and to record the interview was also obtained prior to the interview start. Participants received a pair of movie tickets to thank them for their time.

### Participants

Doctors and advanced care practitioners (i.e., nurse practitioners and physician assistants) who were actively involved in delivering care in the outpatient setting within Atrium Health were eligible to participate. The physician leading the study sent a recruitment letter to clinic medical directors, who then invited their providers to participate in the key informant interviews. Providers were contacted in person by Atrium Health quality improvement coordinators familiar with these individuals through their prior collaborative work. The study team received a list of 24 providers who indicated interest in participating. All 24 individuals received either an email message or phone call. Of these, 17 providers were successfully contacted, agreed to participate, and completed the interview (Table 1). None of the 24 providers refused an invitation to interview; the remaining 7 providers did not respond to emails or phone calls.

**Table 1:** Demographics of providers participating in interviews regarding antimicrobial stewardship (*n* = 17)

Characteristic	Number of providers
Sex	
Male	5
Female	12
Credentials	
MD^a^	10
ACP^b^	7
Practice Setting	
Family Medicine	9
Pediatrics	3
Urgent Care	4
Pediatric Urgent Care	1

^a^Doctor of Medicine

^b^Advanced Care Practitioner

### Data collection

A phenomenological perspective was used in designing a qualitative study to explore providers’ experiences with making decisions about the use of antibiotics in the treatment of patients; the types of factors that motivate their treatment decisions regarding the use of antibiotics; their experiences as a part of a healthcare system in which decisions are made regarding the use of antibiotics; and their perceptions of patient experiences with provider decisions regarding the use of antibiotics to treat their illnesses [22]. Data were collected using semi-structured interviews that allowed for exploration of provider perceptions. The interview guide included questions developed around several areas of interest identified by project stakeholders: a) key factors in antibiotic prescribing; b) communicating with patients about antibiotics; c) helping patients to feel better; d) patient knowledge and experiences; e) the problem of antibiotic resistance; f) barriers to appropriate prescribing; and g) education, training, and reporting. Interviews were conducted on weekdays between the hours of 7:00 a.m. and 5:00 p.m. during November and December 2016 and lasted less than one hour. All providers elected to participate via telephone and all interviews were conducted by the same experienced qualitative researcher. Each interview was audio recorded and transcribed for analysis. Data collection continued until saturation was achieved as indicated by the researcher (TDY) responsible for both conducting the interviews and the primary analysis. To confirm, we conducted two additional interviews with providers beyond our original goal of 15. Data production followed a quality control process whereby transcripts were created by a transcriptionist (KB), quality checked by a trained member of the research team (CDC), and finalized by the original transcriptionist prior to analysis. No identifying information was included in the production of the texts.

### Data analysis

Key areas of focus addressed in the interview guide informed an inductive analysis of the data. Transcripts were initially open-coded at the descriptive level. Second-level coding involved identifying salient categories and condensing initial coding classifications to reflect emerging patterns and relationships among the data. Thematic analysis of the data proceeded from an in vivo (i.e., in their own words) and descriptive level interpretation to more abstracted categories [23]. Written notes taken by the researcher during interviews were also expanded into analytic memos and included as an intermediate component of the analysis process that documented early and emerging interpretations of the data [24]. Major themes were identified based upon patterns evident across provider responses. NVivo Version 10 was used to assist with data management and analysis. Data were analyzed by the same qualitative researcher who conducted all provider interviews.

#### Efforts to ensure quality

As previously described, all participants were employed by one healthcare system in the Southeast United States at the end of 2016, and our study design assumed that these practitioners had experiences with and opinions regarding antibiotic prescribing in the outpatient setting. During interviews with these providers, we frequently asked participants to confirm the researcher’s understanding of their responses. Throughout data analysis and report preparation, portions of the transcript were reviewed and discussed with the primary transcriptionist (KB). In the case of contradicting interpretations, consensus was achieved. Preliminary findings were reported to and discussed internally with the larger research team and key stakeholders, including interview participants. While all data were included for analysis, results presented in this manuscript are excerpts reflecting major themes that emerged. The lead author (TDY), who also served as the interviewer, claims no bias regarding the subject matter.

## Results

Interviews with providers revealed several key elements impacting their discussions with patients regarding appropriate use of antibiotics as well as some primary barriers to reducing inappropriate prescribing. These included patient education and expectations, system-level factors, and time constraints.

### Current antibiotic prescribing practices

To better understand current antibiotic prescribing patterns, we asked providers about key factors they consider when making antibiotic prescribing decisions, how they communicate with patients about antibiotics, and strategies they use to help patients feel better when an antibiotic is not prescribed (Table 2).

**Table 2:** Interview topics, categories, and themes from discussions with outpatient care providers regarding antibiotic prescribing

Interview Topics	Categories	Themes
Key Factors in Decision Making about Prescribing Antibiotics	Acute clinical presentation	Current Antibiotic Prescribing Practices
	Best practices	
	Patient level factors (e.g., age)	
	Workflow	
Communicating with Patients about Antibiotics	Understanding viral and bacterial infections	
	Disease course	
	Symptomatic relief	
	Signs to watch for	
	Follow up	
Helping Patients to Feel Better	Over-the-counter medications	
	Personal care	
	Rest	
Perceptions of Patient Knowledge and Experiences	Expectations are high	Provider Perceptions of Patient Knowledge and Awareness
	Want to feel better	
	A quick fix	
Perceptions of the Problem of Antibiotic Resistance	Largely unaware	
	Some peripheral knowledge	
	Often disassociate	
	Can vary based on demographics	
Perceptions of Barriers to Appropriate Prescribing	Patient education	
	Patient expectations	
	System-level concerns	
	Time constraints	
Education and Training for Providers	System-wide, evidence-based guidelines	Recommendations for Education, Training, and Reporting
	Decision support tools	
Reporting Antibiotic Use to Providers	Multiple forms of delivery (i.e., in person, electronic)	
	Align with current reporting practices	

The providers who participated in this study indicated that they focus on several items including acute clinical presentation (e.g., presence of fever, evidence of bacterial infection); best practices in the form of guidelines and decision support tools; patient level factors such as age, treatment history and social considerations; as well as workflow (i.e., time per patient and patient volume) when making treatment decisions. One physician elaborated on time constraints:

*Definitely. If you’re seeing patients in 10–15 min intervals, it’s very hard. It’s easier to write the prescription for an antibiotic than it is to have the discussion about why they may not need that.* (MD, Interview 10)

Regarding communication with their patients about antibiotics, providers indicated that they often need time to explain the difference between viral and bacterial infections while simultaneously offering measures to relieve symptoms, advising of signs to watch for (e.g., presence of fever, prolonged duration of symptoms) and indicating when and how to follow up if the patient’s condition does not improve. The same provider also shared:

*So I usually try to explain what an antibiotic does because a lot of people don’t understand virus* *versus* *bacteria… Antibiotics themselves are really geared towards bacteria. And if you don’t have [a] bacterial infection, you know, antibiotics [are] not going to help your viral infection at all.* (MD, Interview 10)

Providers aimed to acknowledge and attend to their patients’ feelings while at the same time communicating reasons for not prescribing an antibiotic. The physician summarized:

*When you try to explain to them that what they have is viral and self-limited…and by self-limited I don’t mean shortened. I don’t mean not a pain…or that you don’t feel well, but that an antibiotic is not going to serve any purpose.* (MD, Interview 10)

We also asked providers to describe how they help patients feel better if not prescribing an antibiotic. Providers suggested that in these circumstances they focus discussions on over-the-counter medications that provide symptomatic relief, as well as on rest and personal care (e.g., warm baths and tea) for their patients.

*And [I] just tell them to care for themselves, take a little extra time and care for yourself. Take a hot shower. Take a hot bath. Put your sweat pants on and go home. Take it easy. …I think sometimes people just need that encouragement to take care of themselves a little bit better instead of thinking, well, if I take a Z-pak I can be back at work in 24 h.* (ACP, Interview 4)

### Provider perceptions of patient knowledge and awareness

When asked to share their perceptions of patients’ expectations of when to obtain an antibiotic prescription, providers explained that patient expectations are high for receiving something at their visit to help them feel better.

*They’re showing up in the office. They’re paying for something, and they expect something in return. And my advice isn’t enough.* (MD, Interview 13)

*I think that’s one of the barriers that we have to overcome because patients come into this outpatient setting wanting something. That’s why they spent their money. That’s why they spent their time. And they have an expectation, which we may or may not be able to meet.* (MD, Interview 1)

*     You know, an antibiotic is not a magic pill, which I think a lot of people feel it is.* (ACP, Interview 6)

Moreover, providers characterized patients as being mostly unaware of antibiotic resistance, indicating that some may have only a peripheral knowledge (e.g., they have heard of superbugs).

*I think most patients have a peripheral awareness. I think they know it exists. They know that it could be a problem but they don’t realize how severe, how much resistance is out there. So, they have it in their mind[s]. They hear about it every once in a while but they don’t realize that it’s likely affecting lots of people around them.* (ACP, Interview 5)

Providers further suggested that patients often disassociate themselves from the problem of antibiotic resistance, perceiving it as something that happens to others, but not directly affecting them or their immediate family.

*…there’s a lot of disassociation between the patient and antibiotic resistance. Yeah, but that’s not me. Except for, you know, the Vac-resistant enterococcus and the methicillin-resistant staph. Those are all things that become real problems for people, but only if it’s affecting them at that moment.* (MD, Interview 13)

Finally, providers cited the overall education of the population, health literacy, and socioeconomic status as factors impacting patient awareness of antibiotic resistance.

*I don’t really think that very many people are aware of how significant that problem is. Um, I’m not, I’m not really sure that with the population that I deal with here that is rural, I don’t really think there’s much awareness. In a higher educated population that does their reading, that does their research, that’s, that’s different. …But a lot of folks who are not well educated as far as that, they don’t really understand what resistance is and they don’t understand how devastating that can be until it’s them.* (ACP, Interview 4)

### Barriers to appropriate prescribing of antibiotics

In provider interviews, we specifically asked, “What do you think are some of the primary barriers to reducing inappropriate prescribing of antibiotics?” As follow-up, we also probed for thoughts regarding several distinct elements including patient and provider education and awareness as well as institutional level factors. A key patient level factor identified in provider responses was patients’ expectations for a tangible return on their investment of time and money. One provider described the expectations patients have for immediate relief from their symptoms as follows:

*I think they have a high expectation for an antibiotic. Very high. Very high. I’ve had patients call over the phone: “Can you call me in [an] antibiotic?” “No, I can’t. Not. Nope. Nope because I don’t know what you have.”* (ACP, Interview 4)

Limited provider time with patients was highlighted as a key clinician level factor. Providers explained that they often did not have enough time to spend with a patient in any one visit, even though repeat visits facilitated education around antibiotics over time.

*I need to be seeing patients on an average of every 7 ½ minutes. So it does not give me much time to talk to them about the merits of the treatment plan or what options they have.* (MD, Interview 13)

A lack of patient education regarding the appropriate use of antibiotics was identified as an important education and awareness barrier, and concerns about discontinuity of care as well as patient satisfaction were emphasized as primary institutional level barriers. In terms of education and awareness, providers indicated that most patients do not understand the nature of viral infections, how long the associated symptoms are likely to last, and/or the treatment measures recommended to combat them. This patient level factor creates obstacles to appropriate prescribing.

*I do think a big barrier is patient education. Um, public education. And even just the damage of antibiotics, what we have been doing with antibiotics… I shouldn’t give you an antibiotic because of XYZ but every time I give you an antibiotic and you don’t need one, we’re contributing to this bigger problem. Um and that’s the thing, that you know when someone is not feeling well, they don’t care about the bigger problem.* (MD, Interview 10)

Institutional level barriers that impact antibiotic prescribing practices included concerns about lack of continuity in care and low confidence that different providers would make consistent treatment decisions. Providers mentioned they may see patients only once, especially in urgent care settings. In this scenario, one provider within the healthcare system may not choose antibiotic treatment for a patient, while a different provider may prescribe an antibiotic for the same set of indications.

*Lack of continuity, when you don’t see somebody…all the time, that trust isn’t built. So I think that helps, if you have continuity that helps improve judicious use of antibiotics.* (MD, Interview 3)

*Because they think that you know, they were sick on day three they came in and saw somebody who did the right thing and did not give them an antibiotic and then three days later they’re still sick so they go to urgent care. …And they’re like, “We’re going to go ahead and give you this antibiotic that way it doesn’t turn into something worse.” So it’s a lot of, you know, some of it is being done to ourselves by different colleagues in different situations.* (MD, Interview 10)

Other institutional factors included provider concerns about patient satisfaction and organizational support for their decisions not to prescribe an antibiotic.

*Well the concerns are patient satisfaction is not necessarily quality of care. You’re being judged on what someone’s expectations were when they came in and if they don’t get what they think they should have got, they’re not happy. And that’s gonna affect your patient satisfaction scores. …It’s counter-productive to the whole theory about antibiotic stewardship but that’s part of the thing providers are getting judged on. It’s not quality of care; that’s patient satisfaction.* (MD, Interview 14)

### Recommendations for education, training, and reporting

When asked, “What suggestions, if any, do you have for how to inform clinicians about their use of antibiotics to treat patients,” participating providers indicated that they would be interested in having system-wide, evidence-based guidelines to inform practice and support decision-making. When explicitly asked for suggestions on how to equip providers with current training and educational information regarding appropriate prescribing, providers emphasized a desire for guidelines that would allow them to be “on the same page” with their colleagues.

*I think we do need to have educational training. I think we need to be very clear on the guidelines, evidence-based guidelines on when an antibiotic is appropriate and when it’s not appropriate.* (MD, Interview 8)

They further expressed a desire for decision support tools that would facilitate decision-making at the point of care.

*I am a big proponent of decision support because I don’t think any of us can keep it all in our brains anymore.…Maybe input a couple of criteria regarding your patient’s situation and then it could give you some choices that are all evidence-based but then you would select with your patient the most appropriate option for them.* (ACP, Interview 12)

Beyond promoting consistent and competent care, providers suggested that these resources may empower a provider to confidently deny a request for an unnecessary antibiotic.

*…guidelines that are geared for the providers really liberate the provider[s] to say, “No, I have criteria that says I did the right thing.”* (MD, Interview 15)

Many providers in this study indicated they would like to be informed about their prescribing practices, acknowledging they may not be aware of how often they prescribe when an antibiotic may not be indicated.

*I think if you get really busy you may not even realize how many prescriptions you’re writing in a certain day or a certain week. I mean, I can[not] tell you how many prescriptions I write in any given day, just off the top of my head. So that would be something interesting to see, you know, out of this many patients, this is how many I’m seeing that were acute that I did write an antibiotic on.* (ACP, Interview 6)

Providers recommended delivering information about antibiotic prescribing practices both electronically and in-person, utilizing reporting practices already in place.

*I think it would be helpful to have reporting the way we have with other quality metrics. Have a percentage of times that you have a URI [upper respiratory infection] with antibiotics or bronchitis with antibiotics. And some sort of baseline bar or metric.* (MD, Interview 9)

Of note, while providers were largely in favor of reporting antibiotic prescribing practices, they also expressed concerns about the types of information that would be reported, how widely it would be shared, and what impact it might have upon prescribing behaviors. For example, one provider expressed:

*I don’t know that just telling a provider they gave an antibiotic to a patient is helpful. But maybe if there’s some sort of chart review…it was likely inappropriate in this situation, you could learn from that. But that would require a lot of work.* (ACP, Interview 12)

## Discussion

This study examined provider experiences with antibiotic prescribing and perceptions of ways to reduce inappropriate prescribing of antibiotics in the outpatient setting. Providers indicated that they consider several key factors when deciding whether to administer antibiotics (i.e., clinical presentation, best practices). Within the constraints of appointment times, providers aimed to help their patients find ways to manage symptoms while simultaneously explaining the differences between viral and bacterial infections with respect to appropriate treatments. Additionally, providers noted that patient expectations for relief were high whereas both patient awareness of antibiotic resistance and appropriate use of antibiotics remained low. This lack of awareness was attributed to sociocultural factors along with a tendency for individual patients to disassociate themselves from what they perceived to be a more global issue. Finally, providers identified institutional concerns (e.g., discontinuity of care and patient satisfaction) together with provider time constraints, patient expectations, and a lack of patient education as key barriers to appropriate prescribing.

As in previous research, the results presented here suggest that system-wide, evidence-based guidelines to support decision-making and efforts to improve providers’ awareness of their own prescribing practices would be well received [25]. Consequently, consistent system-level training and prescribing guidelines as well as regular reporting of providers’ antibiotic use may improve levels of appropriate prescribing and has been previously noted in the literature [19, 21, 26]. Educational materials that are concise, easy to access, evidence-based, consistent across practice settings, and utilize both push (e.g., email, text) and pull (e.g., system website) technologies in the dissemination of this information may be most effective. Recommendations for reporting antibiotic use to providers include providing information on a regular basis (e.g., monthly, quarterly), in an easy-to access electronic format. Individual, yet comparative, reporting that remains sensitive in delivery is further proposed and aligns with previous research [27].

Providers recommended patient and public education efforts that occur prior to care delivery (e.g. public service announcements, internet resources, waiting room videos), are reinforced through conversation at patient encounters, and repeated in patient handouts (print outs, pamphlets). This finding is consistent with prior research that suggests that regular and ongoing patient-specific and general public education around appropriate antibiotic use could reduce pressures that providers feel to provide antibiotics [28, 29]. Reducing this burden may in turn have a multiplicative effect as previous research has shown that patient expectation for antibiotics is strongly related to provider overprescribing [30, 31]. Additional recommendations for improving patient education include using multiple media forms, engaging imagery, and language that is easy to understand [32]. One example of this is the Center for Disease Control’s *Get Smart: Know When Antibiotics Work* program [33]. This campaign’s webpage provides materials (e.g., fact sheets, social media messages, graphics, and quizzes) aimed at educating both patients and providers. Moreover, offering tangible items (e.g., tip sheets, symptomatic care kits) to patients that represent a return on their investment may be worth consideration. Consequently, these insights have been used to inform the development of patient education materials, provider scripting, and a reporting dashboard for use at Atrium Health.

Our findings suggest that multiple factors impact antibiotic prescribing practices in the outpatient setting. These include the education and experience of patients, as well as the education and experience of providers [34]. Consistent with prior research, our results also suggest that decision-making is affected by both cultural and system level factors and the dynamics of the patient-provider relationship, influences formerly identified in the literature [35–37]. Research has examined the social context within which prescribing decisions are made, as well as the social norms that guide them, and previously highlighted the impact of these elements on practice [16, 18, 19]. A growing body of literature further suggests that provider characteristics and patient-provider dynamics both impact clinical decision making [34, 35, 38–41]. Thus, a provider’s decision to prescribe can be influenced by numerous factors beyond what is known to be best practice. Any one of these facets, or several in combination, may lead to an increased likelihood of inappropriate prescribing.

### Study limitations

Although multiple coders are preferred in qualitative data analysis, only one researcher coded the data for this study. In addition, all participants in this research were employed by the same healthcare system and their perspectives may not be representative of providers in other settings. Additionally, the results of this study are based on a small convenience sample of providers; this may further limit the generalizability of the findings. Another limitation is that individuals volunteered to participate in the study. Therefore, self-selection bias may have had some impact on the range of attitudes and perceptions provided by the respondents. Notwithstanding, the pressures and challenges related to antibiotic prescribing expressed by providers in this study aligned with those from prior studies in other settings [16–18, 38, 39]. Our convenience sample included primary and urgent care providers. While primary care and urgent care settings have high volumes of antibiotic prescriptions, additional research with providers in other outpatient settings may be useful.

## Conclusion

A myriad of factors including patient expectations and limitations on providers’ time influence providers’ decisions to prescribe antibiotics in the outpatient setting. Our research suggests that both patient and provider education may be key elements of any successful antibiotic stewardship program.

## Abbreviations

Vac-resistant: vancomycin resistant
Z-pak: Zithromax (azithromycin)
